# Professional expectations and patient expectations concerning the development of Artificial Intelligence (AI) for the early diagnosis of Pulmonary Hypertension (PH)

**DOI:** 10.1016/j.jrt.2022.100052

**Published:** 2022-12

**Authors:** Peter Winter, Annamaria Carusi

**Affiliations:** a School of Sociology, Politics and International Studies, University of Bristol, 11 Priory Rd, Bristol, BS8 1TU, UK; b Interchange Research; and Department of Science and Technology Studies, University College London (UCL), 22 Gordon Square, Bloomsbury, London, WC1H 0AW, UK

**Keywords:** Artificial intelligence, Technology development, Innovation, Foresight, Professional, Expectations, Patient expectations

## Abstract

The expectations of professionals working on the development of healthcare Artificial Intelligence (AI) technologies and the patients who will be affected by them have received limited attention. This paper reports on a Foresight Workshop with professionals involved with pulmonary hypertension (PH) and a Focus Group with members of a PH patient group, to discuss expectations of AI development and implementation. We show that while professionals and patients had similar expectations of AI, with respect to the priority of early diagnosis; data risks of privacy and reuse; and responsibility, other expectations differed. One important point of difference was in the attitude toward using AI to point up other potential health problems (in addition to PH). A second difference was in the expectations regarding how much clinical professionals should know about the role of AI in diagnosis. These findings allow us to better prepare for the future by providing a frank appraisal of the complexities of AI development with foresight, and the anxieties of key stakeholders.

## Introduction

Artificial Intelligence (AI) is an emerging technology in healthcare. However, there is uncertainty about how AI will affect the future of healthcare despite significant debate amongst academics, policymakers, patients and the public alike. Common themes in the debate are deskilling (Cabitza et al., 2017; Laï et al., 2020; Nelson et al., 2020), accountability or responsibility issues (Elish, 2018; N. Ipsos MORI, 2017; Lysaght et al., 2019; Ongena et al., 2020a; Ongena et al., 2020b), lack of transparency (Grote & Berens, 2019), data privacy and security (N. Ipsos MORI, 2017; AoMS 2018; Kulkarni, 2019; McCradden et al., 2020a; McCradden et al., 2020b), bias (Challen et al., 2019; Obermeyer et al., 2019), and issues relevant to trust or mistrust (Asan et al., 2020; Jacobs et al., 2021; Lee & Rich, 2021). Whilst there is much apprehension of healthcare AI and, in particular, concerns that AI will replace healthcare professionals (Laï et al., 2020) others argue how AI can ‘augment’ the work of professionals without replacing them (Liew, 2018; Ahuja, 2019). The uncertainty around emerging AI means we do not know, or we are unsure of, how this new innovation landscape will affect the future of healthcare. This means we need to begin asking questions about what this future is going to look like and how it can be shaped responsibly (Brown, Rappert, & Webster, 2000).

To explore the future of healthcare AI, we draw from the sociology of expectations (SE) (Brown, Rappert, & Webster, 2000; Brown and Michael, 2003; Borup et al., 2006). This approach points out how an analysis of expectations puts at the centre of study future visions and their consequences with a focus on innovation and technology. However, this approach to the study of expectations in and around the innovation of healthcare AI has not yet gained attention. This has thrown a spotlight on a crucial dimension of analysis that has been missed in which there has been little critical reflection on the actual dynamics of expectations amongst professionals and patients regarding healthcare AI innovation. This article aims to explore this neglected dimension in order to improve our understanding of the challenges related to emerging healthcare AI so that we can better manage the promises, risks and expectations that they might hold.

We begin with an overview of why it is important to gain a better understanding of the expectations of professionals and patients regarding healthcare AI, outlining the conceptual focus of the article. Section 3 provides contextual information regarding the particular clinical context of this study. Section 4 describes the methods used in running two events, one aimed at professionals, and the other aimed at patients. Both methods explored the expectations of participants regarding how AI is being developed, how it should be implemented, and its implications, impacts or repercussions. Section 5 presents a detailed account of four main themes that emerged from analysis: *AI can result in early diagnosis; data risks of privacy and reuse; responsibility*; and *skilling and deskilling*. The study shows that while professionals and patients have similar expectations of AI, there are also important differences. Points of agreement are the promise and priority of early diagnosis; AI as an assistive technology rather than a decision-making technology; and concerns that over reliance might eventually lead to deskilling. Points where there were notable differences in expectations were the different perceptions of patients being given additional information on other health problems; and the different expectations regarding how much professionals should know or understand about the technologies, and where responsibility ‘kicks in’.

## Expectations, responsible innovation (RI) and foresight

Science and Technology Studies (STS) has a deep and ongoing commitment to the project of exploring the role of expectations in medical innovation, and future healthcare is one in which those expectations come to matter (Brown, Rappert, & Webster, 2000). Drawing on experiences of medical innovation, for example, Brown and Michael (2003) identify how an analysis of expectations can be useful for marshalling resources, coordinating activities and managing uncertainty. Brown and Michael (2003) argue that mobilising the future in real-time or the ability of looking at the future offers us a means by which we can critically analyse the role of expectations in the dynamics of innovation, and in particular how these expectations change and occur over time in accordance to shifting demands and uncertainties. Recent work has explored the ways in which a study of expectations can give way to scenario development and foresight, emphasising in particular the importance of scenario building and the effects of a supposed vision of the future that benefits research institutes and policy making for professional practice (van Lente, 2012).

With the development of healthcare AI in recent years there has been an increasing focus on finding the right balance between the expectations of patients, the public and healthcare professionals in the UK (AoMS, 2018). For example, the Academy of Medical Sciences (2018) have argued that there is a tension between the NHS and commercial developers of AI systems around access to, and the use of, patient data. Meanwhile, others have argued that the acceptance of the AI system that is sought through the process of development is itself a project of expectation, and that the omission of patients in this process may lead to resistance and barriers to implementation (Strohm et al., 2020). As Jermutus et al., (2022) point out, patient expectations of these technologies are made all the more difficult due to the convergence of mixed, even disparate, media and scientific accounts of healthcare AI which influence people's trust in healthcare AI. For example, AI is often communicated in the media as a superior technology with great opportunities and threats that exacerbate inefficiencies and reinforce inequalities (Ciupa, 2017). As Ongena et al., (2020b) argue the increased publication of AI algorithms outperforming radiologists in mammography screening via mainstream media does not necessarily equate to patients having positive expectations about AI systems taking over diagnostic decisions, preferring instead a better “symbiosis” between human experts and AI (see, also Nelson et al., 2020). Other studies on the patient's views of healthcare AI where bits of their expectations emerge in terms of its development and implementation highlighted concerns that wanted resolving, such as conflicting diagnoses between human experts and AI (Nelson et al., 2020), loss of patients’ autonomy and decision making (Esmaeilzadeh, 2020), lack of procedural knowledge on how AI is used (Haan et al., 2019), and lack of transparency about privacy and commercial motives around how their data was used (McCradden et al., 2020a; McCradden et al., 2020b). Lysaght et al. (2019) suggest that as AI systems increase our ability to test and measure, the lack of clarity over how this impacts on the patient-doctor relationship is unclear. This has led some to emphasise that “expectations” from patients and diverse stakeholders are a useful means to surface issues and implications of healthcare AI that may otherwise remain uncovered and little discussed (Laï et al., 2020: 11).

However, much of the debate about the expectations of healthcare AI stakeholders (most of whom are experts in different fields) has approached expectations in a piecemeal manner, with the above research lacking in systematic approaches for identifying expectations (e.g., AoMS, 2018). The SE, in particular, lacks formal and systematic processes for engaging public(s) and stakeholders in discussions on the future of science and technology innovation, and seems to offer only little prospect of transformational change. Stilgoe et al.,’s (2013) framework for responsible innovation (hereafter RI) addresses these shortcomings. In particular, we draw from one of the four principles of the RI framework: ‘anticipation’. Here, the anticipation principle prompts a prospective consideration of the futures that could surround emerging innovation in science and technology. Methods of foresight, technology assessment and scenario development allow forethought about consequences of science and technology development (Eden et al., 2013), and in doing so, lets us anticipate the detrimental implications of new innovations which are often unforeseen, and provides early warnings of future effects (Owen & Pansera, 2019).

Together the SE and RI combine researching the values, needs and expectations of society, with anticipating the broader implications of research and innovation in an ethical, inclusive and responsive way. In this regard, SE shares much with the discourse of RI, but has in contrast emerged to provide us with a number of differences. First, RI provides researchers and organisations with a variety of formal and systematic methods for engaging citizens and stakeholders in the implications of new technologies which are often unforeseen, for instance, prompting researchers to ask ‘what if…?’ questions, considering contingency, what is known, what is likely, plausible or possible (Stilgoe et al., 2013). Second, RI emphasises the need to invite and listen to wider perspectives from publics and diverse stakeholders during the process of innovation, rather than at the end point of innovation (i.e., after these technologies have emerged in society) where discourse around emerging science and technology has become a dominant frame (Owen et al., 2013). This implies that the RI framework, in order to count as responsible, is a flexible process of adaptive learning; learning which can only be achieved by engaging with relevant stakeholders and embedding reflection of their assumptions and practices early on in the development process enacting early societal intervention (Stilgoe et al., 2013).

This article attempts to build a bridge between the field of SE and RI that goes beyond the SE approach that dominates a large part of STS research. Using the anticipation principle as a means of taking into account expectations early on, a new look on how expectations could be collected on the basis of foresight methods is presented in this article. The goal of furthering the integration of aspects of RI into SE expands the methods of foresight beyond asking experts in order to inform how, when and where to intervene. This study attempts to lay the ground for this in the context of AI for early diagnosis in order to see where the convergences and divergences between professionals and patients may lie.

## Developing AI to help diagnose pulmonary hypertension/PH

Diagnosing pulmonary hypertension (PH) remains a major challenge for clinicians, often causing a delay of around two years for a diagnosis (Kiely, Elliot et al., 2013) and causing significant mental and emotional stress to patients (PHA-UK, 2017). A variety of tests or technologies play a key role in PH diagnosis, but are deployed too late in the disease process, and may yield a diagnosis with limited treatment outcomes or poor prognostic markers (Kiely et al., 2013). Unsurprisingly clinical experts around the world are now looking to AI as a route to earlier diagnosis for PH. This is precisely why clinical experts in a UK based Referral Centre for PH are collaborating with AI developers (computer/data scientists) to develop multiple AI systems for these purposes, specifically focusing on Pulmonary Arterial Hypertension (PAH) and one of its sub-categories Idiopathic Pulmonary Arterial Hypertension (IPAH) because they are rapidly progressive and diagnosis is significantly delayed (N. Errington et al., 2021; Kiely et al., 2019a; Swift et al., 2020).

The Referral Centre provides a unique clinical and institutional context with which to explore how these experts collaboratively create their own AI algorithms for the early diagnosis of PH. We saw three AI systems being developed simultaneously for use in the PH Referral centre: the screening algorithm, the imaging algorithm, and the biomarker algorithm, but also collaboratively engaging with each other in bringing to the fore the different stages of an emerging diagnostic process, from screening to diagnosis. Each of these three algorithms are made possible by Machine Learning (ML) techniques. As is often the case with AI development, the screening, imaging, and biomarker algorithms are predictive models and have been typically trained on large and complex healthcare datasets(s). For example, the screening algorithm being developed is for purposes of screening patients ‘at risk’ of PH and is based on a Gradient Boosting Tree approach to help identify patients who have a high frequency of visits across all care settings (A&E, GPs) prior to diagnosis and their relationship between these visits with cardiac respiratory tests, such as heart scans and breathings tests (Bergemann et al., 2018; Kiely et al., 2019a;, Kiely, Lawrie, & Humbert, 2019b). The imaging algorithm is an emerging Tensor-based ML approach that automatically identifies diagnostic features in Pulmonary Arterial Hypertension (PAH) using cardiovascular magnetic resonance images (CMR) (Swift et al., 2020). The biomarker algorithm is in the process of being tested using a variety of ML approaches (such as Random Forest and Gradient Boosting Tree) to help develop classifiers that can distinguish between healthy and diseased biomarkers of the disease (non-pH vs. PAH) in blood samples to be included in the screening algorithm (N. Errington et al., 2021).

Moreover, at this point in the development of the three algorithms it is important to make clear that these are all retrospective studies which use fully curated national electronic health record data (e.g., NHS Hospital Episode Statistics (HES)) and/or locally curated datasets. The use of retrospective data(sets) is the first step in an invaluable and ongoing effort to train and investigate the full potential of the AI algorithm (via initial processes of validation, such as the initial selection of criteria for comparison). Retrospective data is often used to validate the performance (as well as the technical requirements) of the algorithm in controlled laboratory contexts, and often proceeds the prospective application of (real world) data which often comes in the form of a formal clinical trial. This use of retrospective data is very much in alignment with most of the research currently being done on the development of AI in medicine (Nagendran et al., 2020). For more information on the retrospective efforts of the experts in our study, please see (Winter & Carusi, 2022a, Winter & Carusi, 2022b).

## Methods

The Foresight Workshop (hereafter, FW) included a wide range of professionals involved in the development of AI for clinical use. The FW (*n* = 10) comprised of four computer scientists, one biomedical scientist, two clinicians, one consultant nurse, one radiologist, one data scientist, and one professional member of the PHA-UK (a patient group that supports people with PH), representing patient experiences of the condition. The majority of this group were recruited as part of the first objective of the study: to understand the process of developing the screening, imaging, and biomarker algorithms.

The Focus Group (hereafter, FG) (*n* = 11) consisted of six patients living with PH, four carers caring for a loved one with PH, and one member of the PHA-UK (the same one in the FW). Participants were all PHA-UK members and were recruited by publicity through adverts in its Headquarters. Interested participants were then encouraged to discuss their FG participation (voluntary) with a PHA-UK representative who facilitated recruitment. This research was approved by the University of Sheffield Research Ethics Committee on 07/05/2019 (Approval number: 024,923) and included a letter of support from the 10.13039/100002291PHA-UK to show that patients were not recruited via the NHS.

Both FW and FG were conducted to fulfil the second objective of the study: to understand how development and implementation can occur with foresight into future interpersonal, social and ethical effects of the three AI algorithms. Although the two groups were run in slightly different ways (Table 1 and Table 2), they are both concerned with the generation of scenarios and putting participants into the scenario (Eden et al., 2013; Owen et al., 2013). The FW was organised around three scenarios tied to the topics of AI development, integration and implementation, ethics and data justice (Table 1). These scenarios were informed by earlier research we had conducted on the development process of healthcare AI (Winter & Carusi, 2022a, Winter & Carusi, 2022b). The FG was organised around three scenarios tied to the three AI applications being developed for diagnosing PH which combined a discussion of the screening, imaging, and biomarker algorithms (Table 2). What these groups had in common was the objective of revealing scenarios in which people felt comfortable or concerned with ongoing developments of AI for clinical contexts, and the kinds of expectations that were elicited in response to these emerging technologies.

**Table 1:** Foresight scenarios.

**Topics**	**Aim**	**Scenario**	**What if … ? questions**
**Topic 1** AI Development	Explore the role of the professionals in developing AI for PH	Currently we have a situation where there's a very close collaboration between medical practitioners and AI developers. From our fieldwork we saw how important it was for healthcare professional's experience to be a touchstone of truth and also as a safeguard of tolerance.	What if we have a scenario where fewer medical professionals have that experience?
**Topic 2** Integration and the promise of early and more accurate diagnosis	To identify and address any uncertainties and risks related to the integration of AI into the NHS	It is anticipated that AI will become a routine part of clinicians and medical professionals‘ daily lives. From our fieldwork AI will make their work more efficient, accurate and valuable with its integration in clinical teams playing a vital role in accomplishing earlier and more accurate diagnosis.	What if we have a scenario where the integration of AI into the NHS has implications for the NHS, patients and staff?
**Topic 3** Ethics and Justice	To encourage participants to think about the implications these three AI systems might have on ethics and justice	Currently we have a situation where the promising applications of AI systems being developed rely on vast amounts of patient healthcare data. From our fieldwork we saw how datasets were matched or combined with other datasets from a variety of sources that brought challenges to the collection and use of patient data, including potential risks of inequalities in health, such as lack of representation.	What if we have a scenario where ethics procedures and conversations between scientists and clinicians are not included or built into AI development?

**Table 2:** Focus Group scenarios.

**Topics**	**Aims**	**Scenario**	**Questions**
Screening algorithm	Explore patient views on the screening algorithm	AI developers are looking at data to see who will be the patients at risk of developing PH. That information can be used to contact you to come in for further tests with the potential for earlier diagnosis.	How would you feel about the potential for being recalled for further tests after this type of AI application has been used for screening people at risk of PH?
Biomarker algorithm	Explore patient views on the biomarker algorithm	AI developers are looking at blood to see who will be the patients at risk of developing PH *and* other diseases. However, it's not something that you're actually worried about and you might not have any symptoms.	How do you feel about your data being used for that? And maybe, how would you appreciate being recalled for nothing?
Imaging algorithm	Explore patient views on the imaging algorithm	So now you've gone to the clinic and had an MRI scan and AI is interpreting these images. The claims are that AI can recognise patterns in the images and edge doctors towards a diagnosis. Therefore, we might have diagnoses that are informed by AI.	Imagine yourself receiving a diagnosis from your doctor and now instead your doctor might say to you ‘your diagnosis has been made on the basis of AI?’ How do you feel about that? Especially if it informs your treatment.

Both FW and FG lasted around two hours each and were digitally recorded, transcribed, and anonymised. Transcriptions were uploaded to NVIVO 12 to help manage, code and generate themes for ‘thematic analysis’ (Braun & Clarke, 2006). This inductive approach focused on four key themes of expectation which emerged most prevalently in the data. Expectations emerging from the data included: *AI can result in early diagnosis*, early diagnosis outweighs *data risks of privacy and reuse*, and *responsibility lies with specialist clinicians*, and AI will result in *de/skilling* of professionals. These themes were then used to reflect back on the literature as well as the topics that helped guide analysis.

## Results

### AI can result in early diagnosis

The expectation that AI could lead to ‘early diagnosis’ was a common theme between FW and FG participants. Both professionals and patients believed that AI systems held promise for earlier diagnosis. In the FW, the biomedical scientist's statements are typical of the aims of collaborators to achieve earlier diagnosis for PH: *‘one of the points of the project was to try and get to an earlier diagnosis’.* They appeared strongly motivated by empathy with patients (*‘actually what we find is that these people, just want to know what's wrong with them’*). Patients and their carers in this study had been through a long diagnostic journey that included frequent visits to the GP and local hospitals for tests. According to Kiely et al. (2013) PH is diagnosed late: there is often a delay of around two years from onset of symptoms to diagnosis and life expectancy is shortened. Patients on this diagnostic journey are often filled with anger, frustration, confusion and experience incorrect diagnoses (PHA-UK, 2017). In this study FG participants unanimously agreed that AI would be extremely helpful and welcomed the idea that the screening algorithm would reduce the time it took to accomplish a diagnosis for future patients. This is exemplified in the exchange below from FG Participant 7:

There's a period of eighteen months, two years, where there wasn't a diagnosis and that was an absolutely horrendous period looking back on it at the time. And I say that as not the person sitting here, because there was no light at the end of the tunnel. So, it *felt* like you were just being referred around with nobody [Pause] and if this means, what you're saying there, somebody coming behind gets that diagnosis quicker than eighteen months then all of that goes?

**(FG Participant 7, Carer)**

The carer's frustration at the current approach whilst also imagining what this screening algorithm might do to speed up a diagnosis was not unusual amongst FG participants and echoed the FW. For example, professionals in the FW agreed that the screening algorithm was set up to identify people at increased risk of PH and provide some *‘relief’ … to some of those patients who are kind of churning around and not getting a diagnosis*’ (FW Participant 3, Biomedical Scientist). However, FW participants were also acutely aware of what it would be like for patients to receive a referral letter and the fear it might cause (*‘you know this idea of getting a letter saying there's ‘a risk of’ is really worrisome’*: FW Participant 5, Consultant Nurse). Indeed, Foresight participants understood the nature of this challenge and the implications for patients receiving this information. Whilst the unexpectedness of being contacted by referral did not seem to trouble clinician 1 - given how these people were *‘high healthcare resource utilisation users’* - clinician 1 was commited to finding out *‘what information patients want and in what type of way’*. Managing patients’ wellbeing was also at the forefront of this conversation in the broader group too:

What is the right information to give someone before you see them, to say ‘you're at risk?’ What if the clinic is so busy they can't get an appointment for 12 weeks? What is the psychological harm for that person in that 12 week period?

**(FW Participant 2, Data Scientist)**

While FW participants expected AI to be a route to earlier diagnosis, they also articulated some concerns, particularly around the screening algorithm. Their concerns tended to relate to the anxiety over the referral process and the breadth of sensitive yet predictive *‘at risk’* information communicated to them. As other researchers have argued, AI systems must not be relied upon to communicate with patients directly (Schiff & Borenstein, 2019). Rather it raises new questions of emotional, interpersonal and linguistic skills through different types of communication practices as part of a “caring ecology” (Cabitza & Zeitoun, 2019: 161). However, a discrepancy between the two groups emerged when it came to expectations about the disclosure of using AI to patients. Whereas patients expected to be informed about the use of AI in diagnosis, the professionals did not think it was necessary to disclose to patients that decisions about their diagnosis were partially derived from AI (*‘the more we go into telling people how we've collected the [patient] data or made that decision I think the worse that is’*: FW Participant 3, Biomedical Scientist). The professionals likened it to other medical technologies, into which the patient has no more insight than it is a laboratory test or an image (*‘I don't see it any different to a blood test or an x-ray’*: FW Participant 3, Biomedical Scientist). The fact that patients had a different view on this seems to indicate that AI has a different epistemic role for patients than it has for professionals, which differentiates it from other technologies used for diagnosis.

### Data risks of privacy and reuse

The previous section highlighted how professionals and patients had high expectations of AI applications triggering early diagnosis and reducing the negative experiences patients face in the current healthcare system. This next section moves on to analyse the AI developers’ use of patient data behind these innovations and participants’ expectations of privacy issues. FW and FG participants understood patient data as the driving force behind early diagnosis and treatment decisions. However, data risks and implications for patient privacy were narrowly perceived. The privacy concerns of the FG participants were frequently offset by the opportunities it brought for early diagnosis and earlier treatments (McCradden et al., 2020a). For example, one of the FG participants believed that the potential loss of privacy was worth the risk:

There's a unique situation with PH because it's so rare and this is the carer we're talking here. The length of time for finding a diagnosis is in my eyes, *horrendous*. And that's just fact. Whatever they have to give up to make that diagnosis happen quicker is an acceptable cost in my eyes, and I'm the *carer*!

**(FG Participant 7, Carer)**

From the carer's standpoint, the potential loss of privacy so that it can improve the current speed at which a diagnosis is currently accomplished is *‘an acceptable cost in my eyes’.* Although speaking as a *‘carer’*– as in the one whose rights, most notably to privacy would not be infringed upon – the carer's expectations of AI developers having access to their wife's data to drive early diagnosis chimes with the professionals. Similarly, FG participant 5 is not concerned with how their daughter's data is being used as long as it improves early diagnosis of PH. Although they were unaware of the different types of data being used in screening patients at risk of PH, the carer felt reassured that their daughter in this scenario would be called in by a doctor:

It's very positive, I think. Some people might think it's intrusive but when you've been living with the disease and you're back and forth to the hospital, you know, I think it's quite reassuring that the doctors are giving me a call and saying ‘look, there's a problem, come in!’

**(FG Participant 5, Carer)**

Despite the FG stating that less privacy is acceptable for the goal of improving health (McCradden et al., 2020b), they did not fully appreciate how this data was gathered from non-PH patients in the healthcare system and the implications for their privacy.

The second section examines another type of data risk: diagnostic bias in the datasets. Diagnostic biases are judgements and clinical decisions that come from clinical experts, and in which the diagnosis of the expert inevitably contains some degree of bias and becomes encoded in patient datasets (Challen et al., 2019). In the FW, professionals were aware of the diagnostic biases embedded in datasets. However, they also acknowledged how diagnostic bias is part and parcel of algorithm development. Clinician 1, in particular, stressed how the risk of diagnostic bias in a Hospital Episode Statistics (HES) dataset was inevitable and how one must be prepared to live with these types of limitations in the early design stage:

You recognise some bits of the data are not good quality as you alluded to as well. So you have stuff that's very high fidelity or very low fidelity type of information and things you can trust and you can't trust. There are some things I know about that data which are far from ideal but may well be that it represents an *improvement* on what we got at the moment and actually it's never going to be perfect but you can always improve things as you develop them further. So this HES thing, this diagnostic [screening] algorithm, do I think it's good? I think it's probably ‘okay’. It's much better than [Pause] it's a step forward but it needs to evolve over time, so I think you just recognise that nothing is perfect and everything has got its limitations.

**(FW Participant 1, Clinician 1)**

Despite acknowledging how patient data can be *‘high fidelity’* or *‘very low fidelity type of information’* and ultimately *‘far from ideal’* Clinician 1 highlights how it still *‘represents an improvement on what we got at the moment’.* Although FW participants were aware of bias in datasets, it was something that they were willing to put up with as long as they became aware of them and compared datasets with the limitations of other types of diagnostic tools: ‘*but isn't it part of anything whatever tool that you use? I mean it is about recognising those limitations … there's going to be pros and cons with any AI approach so it's very dependant on the user of AI using that AI responsibly’* (FW Participant 1, Clinician 1). In contrast, FG participants were unaware that this type of bias was an issue for algorithm development.

This aspect of bias emphasises the reuse aspect of patient data. In this study the FW were excited at the prospect of using the biomarker algorithm to detect patients at risk of other conditions. The FG, however, were hesitant or even negatively disposed to being told about their risk of developing other conditions. For example, patients and their carers tended to view the biomarker algorithm as a technology that may have unwarranted implications, such as learning about being at risk of other diseases besides PH and subject to further diagnostic tests as articulated by Participant 8, who was diagnosed with PH in 2005:

It would be frightening, wouldn't it, you know? You know somebody could find out you've got this, you've got that, and I think it's alright as it is, you know? I wouldn't want to think that somebody could just say ‘right, we want to see you now because you've got’ [Pause] oh I don't know, some kind of cancer on top of what you've already got.

**(FG Participant 8, Patient)**

For people with PH, finding out about other diseases they may also suffer from is *‘frightening’;* The patient here demonstrates that people living with PH can sometimes feel uneasy at the prospect of knowing more about their physical condition. There is the possibility of learning too much about one's health, and at the wrong time (*‘it would be better to get seen earlier but that's just something else on your mind isn't it?’*). As Klitzman and Chung (2010) observed, people vary in their preferences regarding how much they want to know about a disease, and in some circumstances may not want to know about a disease that has not caused (and may not cause) them harm. The argument that patients should be given a *‘choice’* as a means of empowering them and managing their emotions or psychological aspects of health was brought to the fore by FG Participant 6 (Patient) when asked whether they wanted to know the details of other conditions (*‘or maybe there could be a choice?’*). Furthering their commentary, the patient evoked a future clinical scenario where choices would be offered in a pre-consultation: *‘you decide that ‘yes’ if there's something that they can see that might be a problem ‘yes, I want to know’, or ‘no, I don't want to know’.*

The third type of data risk concerns commercial motives (McCradden et al., 2020a). Although FG Participant 6 was happy about the NHS having access to their data, they felt less assured about the commercial access to their data by external organisations or companies outside the NHS:

I think the other thing we've got to be careful about is that: who else gets access to this data? Because, for example, insurance. If you've got PH and you're going on holiday and you want to buy insurance […] get your chequebook out because it's going to go up!

**(FG Participant 6, Patient)**

FG Participant 6 raises the risk of patient data going outside the NHS (*‘who else gets access to this data?’*), especially when it is shared with organisations such as insurance companies (*‘because, for example, insurance’*). Participant 6 gives an example of how this might open the door for insurance companies to misuse data, and make biased decisions as they pertain to insuring people and prices of insurance policy premiums (Kulkarni, 2019). Reliving a holiday experience, the patient highlights how an insurance company raised the cost of their insurance policy (*‘if you've got* PH *and you're going on holiday and you want to buy insurance […] get your chequebook out because it's going to go up’*) because they were able to check whether they did or did not have any underlying health condition (*‘because they can check to see’*). In telling their story, Participant 6 points out how despite not being prescribed oxygen for the plane, their prescription for the use of *‘oxygen at home’* nevertheless raised their holiday insurance premium substantially: *‘my insurance went from £300 to £800 just for me alone!’.*

However, many of the participants had not considered how much of their personal data was ‘out there’ or what it was being used for and chimes with the findings of privacy concerns of health data elsewhere (N. Ipsos MORI, 2017; AoMS, 2018; McCradden et al., 2020a). Some had not grasped the potential financial value of their data and were naïve to the possibility of misuse. When the FG was asked whether they were happy that their data could be sold by the NHS to foreign companies and used for more *‘corporate purposes’*, FG Participant 8 insisted how corporate companies’ involvement only had the patient's best interests at heart:

I'm sure they won't do anything to you purposefully. You know you might give them some information about something that's wrong with you but they're only going to want that aren't they to try and help anybody else, I'm sure?

(FG Participant 8, PH Patient)

Participant 8′s comment turned the conversation towards the USA's insurance-based health system and how insurance companies who have access to this data are likely to base their policies on risk selection so that they can remain profit making entities (Kulkarni, 2019). The idea that the fate or health of the individual operated under a profit motive did not sit easy with the group. In doing so, questions were raised around who benefits from patient data and profit motives which opened up room for a PHA-UK Representative to state how they had been approached by pharmaceutical companies on several occasions (*‘we get contacted an awful lot by pharmaceutical companies. Now they see your data as absolute gold dust!’*) to sell their data for profit (*‘it would be worth millions! Hundreds of millions of pounds!’*: FW Participant 11, PHA-UK Representative). The moment when Participant 11 revealed how *‘insurance, pharmaceutical companies they've got a huge stake in your data’,* highlighted the breadth and complexity of data risks: it can ask questions about the access of patient data by different actors; the reuse of patient data; it can pursue questions about the misuse or misappropriation of patient data; it can highlight ways in which patient data can have significant economic value; and it can draw attention to how patients can be paid for their data.

### Responsibility lies with specialist clinicians

Previous research has shown how there are different views on who should be held responsible for AI systems (Elish, 2018; N. Ipsos MORI, 2017; Lysaght et al., 2019; Ongena et al., 2020a; Ongena et al., 2020b), especially when machine learning goes wrong (N. Ipsos MORI, 2017). In these and other cases, participants expressed concerns about the unresolved question of responsibility and the interpretation of outputs from the AI application. In this study both FW and FG participants wanted specialist PH clinicians to have ultimate responsibility over the custody and control of AI, including the interpretation of outputs. This observation suggested that the expectation of responsibility was tied to the specialist expertise of the clinician and where their experience of diagnosing PH was seen as vital in using the technology.

In the context of the screening algorithm, both FW and FG participants highlighted their concerns about *‘at risk’* patients being referred to non-specialist clinicians. Whilst the screening algorithm brought with it a sense that it was quicker than the existing approach for detecting and referring patients *‘at risk’* of PH, both groups argued that it only added value to diagnosis in the context of specialist expertise in PH. This pointed to how the screening algorithm was only useful if it referred patients to a specialist centre, so that they could be examined by a clinician with specialist knowledge of PH rather than being referred to a GP or a local Hospital. For example, FG participant 5 was happy that such an algorithm would have detected their daughter's health seeking pattern as being ‘at risk’ of PH, however, they felt it would only have been of value if their daughter was then referred to a specialist centre. This is highlighted in the quote from Participant 5 whose daughter had been seen by non-PH specialists in [anonymised General Hospital] and who had a suspected but undiagnosed heart problem:

They were in and out of Hospital in [anonymised General Hospital] so they were checking their heart thinking they had a valve problem, they had a heart murmur, their heart was enlarged and it wasn't until [anonymised General Hospital] a year later sent them to the [anonymised PH clinic] that we actually realised it was Pulmonary Hypertension. […] But I think it was just the fact that [anonymised General Hospital] isn't a specialist centre so they would not have flagged up on something like that until they've said ‘we've done what we can, maybe you should go and see the [anonymised PH clinic]’ and that's when we went there that they actually started to investigate PH. […] In [anonymised General Hospital] they might see a list of symptoms and think: ‘Oh, it might be a heart problem?’ but [anonymised PH clinic] might see the same and just go: ‘that's PH!’

**(FG Participant 5, Carer)**

Despite a very frustrating experience and late diagnosis Participant 5′s desire for specialist expertise was mirrored by the professionals in the FW. Diagnosis needed to be accomplished by a specialist or rather, a team of expert PH clinicians to either confirm or rule out the disease. A data scientist collaborating with a specialist clinician in developing the screening algorithm pointed out that the purpose of the algorithm was to make sure that these patients would be able to see a specialist *‘either a little bit earlier or at the right time … who then takes it from there’*. PH clinician 1 expands on this:

I think one of the values of people who are high healthcare resource utilisation users is to get them in some form of clinic with somebody who can actually have a bit of a look at them to try and work out what the problem is. So I see it as a potentially very beneficial thing that these guys have clearly got *some* sort of problem where they're having multiple tests and investigations done, so maybe the idea is to try and signpost them to an individual who might be more likely to be able to make a diagnosis in a specialist centre.

**(Participant 1, Clinician 1)**

Despite the promise of the AI identifying ‘at risk’ patients clinician 1 also felt that this would benefit patients by making sure they are referred to PH specialists who are expert at diagnosing respiratory problems. In these instances, both professionals and patients were attuned to the importance of specialist centres and lead clinicians.

This expectation that specialists should have responsibility for allowing the AI to have a role in diagnosis was seen as analogous with buying a self-driving car (not just travelling in one). FG participants unanimously agreed that the responsibility for the driverless car remained the owner's alone. Regarding if the self-driverless car *‘ran somebody over’*, FG Participant 8 (patient) noted *‘but you've got to be responsible if you buy that car?’.* The ensuing conversation amongst other members of the FG seemed to confirm this view, and extended to the moral implications of crashes and accidents, FG Participant 1 (patient) stated *‘if it's your car, it's your responsibility’.* On this analogy, everyone agreed that specialist human clinicians should be held responsible for diagnosis or misdiagnosis, and not the AI. This is reflected by FG Participant 5 whose daughter was misdiagnosed late, in the context of a question about who had responsibility for misdiagnosis if AI was to be included in the diagnostic mix: *‘it has to be the human's responsibility because AI is just an aid, it's like a piece of software. You know you can't hold it responsible’.* In this way Participant 5 is proposing that AI functions as an assistant to the clinician.

Both FW and FG participants saw the AI as a tool or resource to inform diagnostic decisions, indicating that the acceptability of AI seems to depend on it having (only) an assistive role in the diagnostic process. It was anticipated that the AI would assist the clinician's specialist expertise only and not reduce a clinician's capacity to take control when the situation demands. Both FW and FG participants did not expect the AI to make a final decision as to whether a patient did or did not have PH. For example, the biomedical scientist working on both screening and biomarker algorithms regarded each algorithm as just another test or investigation that was built on top of their diagnostic toolkit (*‘is that any different to any other technology or any other advancement?*’) and assisted clinicians (*‘we're not talking about using AI to develop a ‘yes/no’ we're talking about developing something that gives assistance to the person making a decision’*).

Professionals also stress the use of AI to make small-step decisions. According to Foresight participants, the algorithm's role in making small-step decisions played a critical role in forming and forging the acceptance of AI amongst adopters. This incremental approach not only served as a base towards more concrete decisions, but also to downplay the epistemological presumptions about the promise of AI (Elish, 2018). The data scientist talks about the way in which AI should be used to provide clinicians with small-step decisions on which other decisions can be formed in response to a question about how clinicians might learn to accept the AI:

I think starting smaller would help? I think a lot of the time AI practitioners promise the world and they come in with this massive hype and there's talk about you know almost replacing people and automating jobs and things like that. But I think if you think about the NHS and again the project that we're doing we're so many steps before a clinical decision is made and you're just hopefully getting a person either a little bit earlier or at the right time to see a specialist who then takes it from there. Maybe that feels like a better place to start?

**(FW Participant 2, Data Scientist)**

There was agreement regarding small-step decisions and the ways in which outputs from AI would be discussed with other colleagues, such as in multidisciplinary teams (MDTs), and the potential of output data to inform clinical decision making around PH related tests/investigations. Professionals knew that none of the three algorithms could offer certainty about the patient's condition. It was expected, however, that one or all of them may be one of the steps towards earlier decision making. For example, the imaging algorithm was expected to be useful in problem flagging and triaging patients with unusual or suspicious findings. Even when the algorithm is said to highlight a potential feature of PH, the radiologist would inspect the algorithm's output *‘so it has some kind of human oversight’* yet stop short of making a definitive diagnosis because the output is essentially restricted to *‘sort of blobs on an image’* (FW Participant 4, Radiologist).

The FG also assumed that all three AI systems would have an assistive role in diagnostic practice and expected that small-step decisions would drive diagnosis. FG Participant 6, for example, liked the idea of the imaging algorithm assisting the radiologist, but was hesitant about the idea of it replacing the radiologist:

I wouldn't want it to take over from the clinician […]. I understand that the AI would be better at looking at thousands and thousands of images quickly where a clinician would take hours to look through all this. I want it to be flagged up that this is a problem: ‘we think that this is a problem by the AI’ but then to be looked at by a clinician who can then say ‘yes’ or ‘no’. But the difficulty there is if the clinician is not seeing the small changes that the AI is picking up?

**(FG Participant 6, Patient)**

While the patient clearly outlines their expectation of the imaging algorithm as an assistive tool (helping with more time-consuming tasks and flagging potential issues), they also felt it may detect something the clinician was unable to see or understand (*‘the difficulty there is if the clinician is not seeing the small changes that the AI is picking up’*). This finding touches upon the distinction between skilling and deskilling made by Liew (2018) and Ahuja (2019) and is something that will be explored in more depth in the final section of this article.

### Skilling and deskilling

Despite the fact that future visions of AI assisted diagnosis were expected to be conducted by PH specialists, questions were raised by FW and FG about whether specialist clinicians had to learn new skills in order to interpret or explain new characteristics of PH. This aligns with recent research conducted amongst radiologists (Liew, 2018; Ahuja, 2019). A specialist PH clinician involved in interpreting the training outputs of the imaging algorithm related how his observation of a new mathematical measure for PH (as one of its outputs) did not strongly correlate with what he already knew about the disease:

It was a measure that we all recognise that's reasonably showing it as the one that obviously correlated well with the clinical outcome but didn't seem to correlate with the logical understanding of the pathology? And there's this really interesting tension thing [and] if you start to see a piece of big data showing up an algorithm and the algorithm makes some association, and that association isn't something that you can map onto your understanding of the disease process. So you have to ask two questions: either, do I not understand the disease process and is it telling me something interesting about the disease? Or, alternatively, does the algorithm measure some form of surrogate and in which case can I trust its maths?

**(FW Participant 6, Clinician 2)**

While clinician 2 found value in the imaging algorithm detecting a potential new feature of PH, they were uncertain of the training outputs that *‘didn't seem to correlate with the logical understanding of the pathology?’*. At this early stage in the development process, the two clinicians, radiologist and biomedical scientist were sometimes confused by the associations that their algorithms would produce that did not seem to fit well with their prior understanding of PH. In the case of the imaging algorithm, the radiologist highlighted how it sometimes produced unexpected outputs or unfamiliar associations:

If you do a simple regression which is a bit like a very fundamental type of machine learning where you just try and add measurements together sometimes it will just throw up something which is not what you expect and that could be because of underfitting, it could be you haven't got enough data [it could be true of course]. You know it could be working out whether actually you can trust that. Those are the findings and interrogating it and that's why the machine learning we're doing is quite good because [in terms] of *visualisation* and interpretability, you can start to see in the image where it's actually telling you that these are something [suspicious]

**(FW Participant 4, Radiologist)**

The radiologist indicates how the AI has the potential to produce outputs that do not resonate with his current understanding of PH (*‘it will just throw up something which is not what you expect’*). Yet this uncertainty is somewhat mitigated by producing outputs that are both visual and interpretable (*‘the machine learning we're doing is quite good because of visualisation and interpretability’*). It also serves to demonstrate how their understanding of PH and the logics behind regression (including problems such as *‘underfitting’*) are inherently tied to his interpretation. As the radiologist constitutes the interpretation of PH in new ways, a shift is enabled in the understanding of disease highlighting AI developments role in the skilling and ‘augmenting’ debate initiated by commentators on healthcare AI (Liew, 2018; Ahuja, 2019).

The imaging algorithm offered professionals important information, but as illustrated in clinician 2′s quotation, this will likely require three components of skilling. First, there are the traditional skills of acquiring specialist knowledge of PH (*‘do I not understand the disease process’*), second there is the skill of judging the extent to which the AI system is exposing different aspects of the disease (‘*is it telling me something interesting about the disease’),* and third whether it can be trusted (*‘does the algorithm measure some form of surrogate and in which case can I trust its maths?’*). The complex skills of working with AI require knowing when to fall back on established knowledge about and experience of the disease, and knowing when to have that knowledge modulated in some way by the AI. The biomedical scientist in particular pointed out the issue of outputs that are unusual but may still be relevant (*‘we don't have very good ways of picking up unknown-unknowns’)* which he also advocated as one of the promises of AI ‘*it may well be that's just a weird thing that seems to correlate with something that isn't weird that makes sense of the pathology or it could be simply some new insight into [the disease]’.*

This matter of skilling was also closely tied to a concern of deskilling as hinted above by FG Participant 6 *(‘the difficulty there is if the clinician is not seeing the small changes that the AI is picking up’).* On the topic of AI development, the radiologist was asked what the future would look like for the imaging algorithm and how having such an assistive technology for detecting disease would work in the future with junior radiologists. This was a moment where the radiologist felt that the AI was likely to perform better than junior radiologists:

I guess it gets to the point where it's seen so much that it's better than a junior radiologist who is just starting out, because they're trying to develop that wider experience. Everybody sub-specialises *as well* so you're always seeing things that are outside of your expertise. *But* yes, […] I guess that the AI would perform better than non-specialist radiologists. The risk is overreliance and we become more reliant on the AI rather than sort of just looking at the AI as an aid. The danger is that it becomes so good that you start to trust it more and more and less and less your own skill. Not for the current generation but for the next generation.

**(FW Participant 4, Radiologist)**

Anticipating the future of the imaging algorithm, the radiologist stressed the importance not just of the algorithm becoming better than junior radiologists but also the radiologist becoming deskilled due to overreliance on the algorithm (Cabitza et al., 2017; Laï et al., 2020; Nelson et al., 2020). This point often referred to as ‘automation bias’ (Cabitza & Zeitoun, 2019: 3) was expanded upon by a computer scientist:

That is what is happening with airplanes at the moment because the system is so good at flying that all the major accidents […] are always due to the fact that people were *deskilled* and the moment there was a situation where the instruments were saying the wrong thing or presenting an unusual thing they always took the wrong decision and the plane fell down from the sky.

**(FW Participant 7, Computer Scientist)**

Whilst this does not provide immediate tensions or concerns with the current specialists, it raises the concern that some skills may be lost in the AI transition. This raises the question as to how to mitigate against overreliance of AI systems and deskilling of experts in the future (Laï et al., 2020).

In the FG, the matter of overreliance and its effect on deskilling was also raised. For example, Participants 6 and 7 were concerned about clinicians becoming overreliant on AI assistance and not being able to give an account of the role of AI in arriving at a diagnosis:

**FG Participant 6:** I just worry that you can't interrogate it but as a *clinician*, maybe that's just me, but as a clinician I would want to know how it's come to a decision.

**FG Participant 7:** You want to know why it did it!

**FG Participant 8:** That's because you're anal, you want to know everything!

**FG Participant 6:** But I want to know why? How would I learn as a clinician if this AI was taking over all this diagnosis? How would I *learn* and grow as a clinician if I couldn't interrogate it?

Participant 6 is worried about the clinicians becoming deskilled. Their concern is that clinicians would lose their authority over the technology, how developing a specialist medical knowledge would be stunted by the technology, and how expertise with which to engage and *‘interrogate’* the technology would be lost.

This is in contrast to the FW. Clinician 1 felt that needing to understand how the AI came to a decision was unnecessary:

I wouldn't really feel necessarily I absolutely need to know *everything*. I think if you're in that sort of process where you need to understand and be comfortable with every single part of that sort of process [Pause] the way that we work as humans can make that process very long winded and ending up doing multiple different tests and investigations which if you're able, as Participant 6 talked about, it's about managing uncertainty, being able to manage a degree of uncertainty.

**(FW Participant 1, Clinician 1)**

Participants in this scenario suggest a complex picture: there appears to be a tension between professionals and patients in terms of understanding the technical points of the AI system for both diagnosis and patient communication. FW analysis suggests that this tension may reflect the taken for grantedness of the development process to which the professionals are embedded. This is an important point - importantly, we must remember that clinician 1 is *embedded* in the development process and their views come from a highly situated vantage point that is said to build trust (Winter & Carusi, 2022a) and ‘de-trouble’ transparency issues (Winter & Carusi, 2022b). Clinician 1 continues:

I wouldn't have a concern I think if there was a really good algorithm that was a really good diagnostic and I had seen it in action a few times and then you know you could then adopt that and feel fairly comfortable with it. I don't think I would necessarily have to have years of experience using it *or* understanding exactly how it *works,* but if I was able to *test* it in some type of way and it appeared to be fairly accurate, I would probably feel quite comfortable with it.

**(FW Participant 1, Clinician 1)**

Here, we must ask: would clinician 1 have had the same view (i.e. not needing to understand how the machine came to a decision/output) if he had not been part of the development process? This section again reinforces not only the professionals’ participation in the development process and professionals’ acceptance of the algorithm it also highlights its educational effects; an educational space for technical knowledge that mediates the interpretation and communication of outputs.

## Discussion

One of the most significant challenges facing professionals’ implementation of healthcare AI is making sure that the AI is accepted by both patient and public (Haan et al., 2019; Ongena et al., 2020b). This means AI must be developed in a responsible manner, not only ensuring that it is balanced and representative of people whose data is captured, but also making sure the patient and public have a voice in both its development and implementation (N. Ipsos MORI, 2017; AoMS, 2018; Laï et al., 2020). By examining the expectations of professionals involved in developing three AI systems for the early diagnosis of PH, and of patients with experience of the diagnostic process and disease, this article has sought to offer an original contribution to existing work on STS, RI, and the SoE. With this article, we contribute to studies of expectations by expanding the methods of foresight beyond asking experts, by which expectations of the future are created in structured forms, such as in the case of scenarios.

In this article we have discussed four shared expectations of professionals and patients in the early stages of innovation: the first expectation is that *AI can result in earlier diagnosis,* a reading that resulted in participants emphasising how early diagnosis was a priority. The second expectation, however, demonstrated how this priority for a diagnosis was worth some *data risks of privacy and reuse*. Data risks were narrowly perceived by both groups: whereas patients had a relative lack of knowledge on privacy and stewardship of their data, professionals were somewhat unconcerned about bias because of how early they were in the development process. The third expectation demonstrated the *responsibility* of specialist clinicians, especially in ensuring that the AI remained having only an assistive role in diagnosis. Existing research on responsibility has tended to concentrate on the extent to which AI can deskill healthcare professionals (Laï et al., 2020), yet we show how professionals engaged in the development process are entwined with the technology and learn a new skill set by way of the logics of computer science.

However, our data suggests that there can also be disparities between what professionals and patients want. Although professionals found promise in AI offering further insights into new diseases, patients were hesitant or even negatively disposed to being told about other diseases on top of their condition. Patients seemed also to expect a higher level of understanding of the technologies on the part of the clinicians, than the clinicians had of themselves. Further research is needed in order to get a clearer understanding of this discrepancy. This latter issue is extremely important in terms of preparing the future NHS workforce for these technologies, both in terms of skills and behaviours around healthcare AI (AoMS, 2018). Highlighting these disparities between professionals and patients then reminds us that more work needs to be done, including the role expectations play, to ensure that emerging healthcare AI is opened up to a wider range of stakeholders in order to minimise risks or prepare for concerns relating to its use. It is important to note that in this article our aim is not simply to analyse expectations to look into the future, but to identify the variety of relevant expectations as evidence for possible practical intervention. In this instance, the AI in the Clinic project was instrumental in raising potential implications and patient expectations with regard to research and innovation of the three AI algorithms being developed, and has since influenced the decision to include the views of patients in the type of referral system that is needed to inform them of potential PH obtained as part of the screening program.

## Conclusion

In this article, we have analysed the expectations of professionals and patients in relation to the development and future implementation of three AI systems for diagnosing PH. As healthcare AI comes to occupy a significant position within clinical contexts, and the ‘promise’ of AI becomes more prevalent in everyday discourse, there is a lack of research on what may confront clinicians and patients in these contexts. Therefore, it is increasingly important we turn more attention to these key stakeholders in order to prepare and challenge the promissory and future-orientated practices of tomorrow. By focusing on the role of expectations in healthcare AI, this article offers a novel contribution to this existing focus.

However, these more promissory visions (as in the sociology of expectations) and the study of knowledge practices (conceptualised as styles of anticipation) come with the difficulty of opening up AI innovation with patients and the general public, meaning researchers may not necessarily provide the ideal entry-level knowledge or techniques for patients who may be unfamiliar with healthcare AI subjects (a matter made all the more challenging in times of national crisis, such as Covid-19, cost of living, and climate change). Similarly, this article does not address the epistemological or methodological challenges related to engaging patients in healthcare AI systems that do not yet exist. These two aspects are important to note, especially in relation to processes that may engender greater inclusion. For example, as we saw in this article, members of the public were included in our research, but they were family members of the PH patients and did not object to the use of their personal healthcare data being used for PH and other healthcare AI related research. Going forward, it is worth considering including other voices (different patients and different members of the public) in discussions of PH-related AI systems.

Such a perspective presents a roadmap for further research. First, future research needs to look at the AI system being developed for a specific patient group (or disease area, like PH) and its relationship to wider medicine that collects and repurposes relevant patient/public data. E.g., how do researchers extend their participant recruitment to include other patients or publics whose data is also part of the training data for ML and will in the end have been considered for the end product? Second, if more research allows the inclusion of a diverse range of patients, publics and/or marginalised voices to emerge there needs to be more work on the exploration of methods for inclusion to address the future impacts of AI systems (see, for example, de Saille et al., 2022). Therefore, a third issue is how to operationalise methods to help researchers communicate and engage the abstract nature of healthcare AI research (from development to implementation) to patients and the wider public, especially of patients with diverse racial, ethnic, and socioeconomic backgrounds? E.g., illustrations, photo voice, and virtual/augmented reality for wayfinding in unfamiliar clinical environments, such as future clinical consultations that are in the process of integrating AI-based devices. This would then also need to involve empirical investigations on future patient-healthcare professional relationships and clinical contexts that need to adapt to the new technology. For example, how does the integration of healthcare AI produce, reproduce, and disrupt relations, good or otherwise, between healthcare professionals, patients, technologies, fields of expertise, and institutions? The task is huge considering the promises and perils involved in healthcare AI systems. Nevertheless, the complex task ahead of us can also be rewarding, creating new possibilities for future research and prefigures more responsible technology.

## Ethics approval

This study involves human participants and was approved by the University of Sheffield Research Ethics Committee on 07 May 2019 (application reference number: 024,923). Participants gave informed consent to participate in the study before taking part.

## Declaration of Competing Interest

The authors declare that they have no known competing financial interests or personal relationships that could have appeared to influence the work reported in this paper.
